# Structure and function of the *Nppa*–*Nppb* cluster locus during heart development and disease

**DOI:** 10.1007/s00018-017-2737-0

**Published:** 2018-01-04

**Authors:** Joyce Man, Phil Barnett, Vincent M. Christoffels

**Affiliations:** Department of Medical Biology, Academic Medical Center, University of Amsterdam, Meibergdreef 15, 1105 AZ Amsterdam, The Netherlands

**Keywords:** Atrial and brain natriuretic peptide, Epigenetics, Gene cluster, Heart development, Heart regeneration, Hypertrophy, Super enhancer

## Abstract

Atrial natriuretic factor and brain natriuretic peptide are two important biomarkers in clinical cardiology. These two natriuretic peptide hormones are encoded by the paralogous genes *Nppa* and *Nppb*, which are evolutionary conserved. Both genes are predominantly expressed by the heart muscle during the embryonic and fetal stages, and in particular *Nppa* expression is strongly reduced in the ventricles after birth. Upon cardiac stress, *Nppa* and *Nppb* are strongly upregulated in the ventricular myocardium. Much is known about the molecular and physiological ques inducing *Nppa* and *Nppb* expression; however, the transcriptional regulatory mechanisms of the *Nppa*–*Nppb* cluster in vivo has proven to be quite complex and is not well understood. In this review, we will provide recent insights into the dynamic and complex regulation of *Nppa* and *Nppb* during heart development and hypertrophy, and the association of this gene cluster with the cardiomyocyte-intrinsic program of heart regeneration.

## Introduction

Pathological stress in the heart results in physiological changes accompanied by alterations at both the transcriptional and epigenetic level. These stresses include cardiac hypertrophy and ischemic injury (myocardial infarction). During hypertrophy, the myocardium undergoes adverse structural remodeling that can lead to heart failure, the heart being unable to meet the circulatory demands of the body [1]. Myocardial infarction leads to loss of muscle mass, scar formation and compensatory hypertrophy [2]. A commonly observed response during cardiac hypertrophy is reactivation of the “fetal gene program”. Normally, these fetal genes are abundantly expressed in the prenatal heart but become downregulated after birth. Once the heart undergoes pathological stress, the expression of these genes is induced and this response is thought to play a role in the process of cardiac remodeling and compensation [3–6]. The induction, however, may be orchestrated by a stress-induced regulatory mechanism different to that of the developmental regulatory program [7, 8].

*Nppa* and *Nppb*, cardiac genes encoding atrial natriuretic factor (ANF) and brain natriuretic peptide (BNP), respectively, belong to this fetal gene program. Both genes are abundantly expressed in the atrial and ventricular myocardium during embryonic and fetal stages. After birth, both genes remain expressed in the heart, however, postnatal expression of *Nppa* is strongly downregulated in the ventricles [9–11]. Upon stress, the pro-peptides are released by the heart and the ventricular expression of both *Nppa* and *Nppb* is strongly increased in the cardiomyocytes [12, 13]. Because of this feature, the gene products, especially NT-pro-BNP that has a longer half-life compared to BNP and ANF, serve as reliable molecular markers to assess cardiac disease and heart failure progression [14, 15]. Additionally, *Nppa* has also become an important marker for myocardial chamber differentiation and congenital heart defects [16, 17]. The importance of *Nppa* and *Nppb* in heart development and disease has initiated in-depth studies on the transcriptional regulatory mechanisms of these genes. Insights into these mechanisms have already substantially increased our understanding of the molecular events underlying heart development and pathological stress of the heart [18].

The paralogous genes *Nppa* and *Nppb* are positioned in close proximity to each other and organized in an evolutionary conserved gene cluster [19–21]. The structural organization and regulation of *Nppa* and *Nppb* expression have proven to be more complex than was initially thought [7, 8, 22–24]. Therefore, the identification and functional analysis of regulatory sequences of the *Nppa*–*Nppb* cluster has been challenging. Nevertheless, current genomic technologies applied to study epigenetic landscapes, chromatin structure and gene regulation (e.g. chromatin immunoprecipitation sequencing and chromatin conformation capturing combined with transgenic reporter mice) has shed light on the regulatory mechanisms of the *Nppa*–*Nppb* cluster in vivo [8, 13, 23, 24]. In this review, we will discuss recent progress in deciphering the regulatory landscape of the *Nppa*–*Nppb* cluster during heart development and disease.

## Gene clusters: conceptual framework of sharing and co-regulation

The spatial and temporal pattern of gene expression is regulated through *cis*-regulatory DNA elements (e.g. promoters, enhancers, insulators, repressors) that function in strictly context-dependent manners. The transcriptional machinery that drives cell-specific gene expression involves the binding of transcription factors and co-factors at specific locations on the DNA via sequence-dependent affinity. This process is coordinated by epigenetic motifs and signatures, and the three-dimensional arrangement of chromatin, which is responsible for bringing necessary components in spatial proximity [18, 25–27]. During evolution, the natriuretic peptide genes *Nppa* and *Nppb* have arisen from the ancestral CNP-3 gene through the process of gene duplication followed by divergence [28]. *Nppa* and *Nppb* are positioned in close proximity to each other in the mammalian genome, separated by only several kilo base pairs (kbp) of DNA sequence. Comparative studies have demonstrated that these paralogous genes show very similar expression patterns in the developing atrial and ventricular chamber myocardium of mouse, rat and human. In contrast, birds have lost the *Nppa* gene, and their *Nppb* gene is expressed at high levels in both atria and ventricles [9, 29]. Both *Nppa* and *Nppb* are upregulated in response to hypertrophy [30] and in the injury border zone after myocardial infarction [13]. These common features of *Nppa* and *Nppb* suggest that this gene cluster may contain *cis*-regulatory sequences shared by both genes, and a topology that facilitates co-regulation during development and stress. Sharing of regulatory sequences and co-regulation of clustered paralogous genes has been proposed previously; however, to date only few examples have been comprehensively described, including the *Iroquois* (*Irx*) and *Hox* gene clusters.

The *Irx* gene cluster is present in both invertebrates and vertebrates. In mammals, the *Irx* genes are divided into two paralogous clusters, *IrxA* (*Irx1*, *Irx2* and *Irx4*) and *IrxB* (*Irx3*, *Irx5* and *Irx6*), located on different chromosomes [31]. In both clusters, the orientation of the three genes is strictly conserved and organized. The developmental expression patterns of clustered genes *Irx1* and *Irx2*, and of *Irx3* and *Irx5*, respectively, are highly similar [32]. All six genes are expressed in specific patterns in the heart, and *Irx3*, *4* and *5* are involved in cardiac development and conduction [33, 34]. Extensive screening of the genomic regions of the *IrxA* and *IrxB* cluster revealed highly conserved non-coding regions with *cis*-regulatory elements. These *cis*-regulatory elements physically interact with the promoters of the first two genes of the *Irx* gene clusters. Furthermore, *Irx1/Irx2* and *Irx3/Irx5* are engaged in promoter–promoter interaction and this explains why their expression patterns overlap during development. The third genes *Irx4* and *Irx6*, respectively, do not seem to interact with the other two genes of their cluster or their shared regulatory elements and consistently show distinct expression patterns [35].

*Hox* genes play a crucial role in vertebrate anterior–posterior patterning and limb development [36–38]. In mammals, 39 *Hox* genes are found organized in four genomic clusters (*HoxA*, *B*, *C* and *D*) that are localized on different chromosomes. The regulation of *Hox* genes is controlled by shared, distant regulatory regions. Moreover, the epigenetic state and chromatin organization of the *Hox* clusters determine the function of regulatory elements in the regulation of the *Hox* genes [39, 40]. The regulation of *HoxD* cluster during limb development has been shown to be tightly controlled by a collection of regulatory elements distributed over two gene deserts (a regulatory archipelago) on either side of the *HoxD* cluster. Through conformational changes in the *HoxD* locus, these regulatory elements are brought together to regulate *HoxD* gene transcription and coordinate the transition from early to late limb development [41–43].

The examples of the *Irx* and *Hox* gene clusters provide a conceptual framework for co-regulation by shared *cis*-regulatory elements in the locus or even at long distance. It has been proposed that the structural stability of these clusters throughout evolution is maintained by the sharing of conserved regulatory elements by the genes within the cluster [44]. More recently, other loci harboring clustered paralogous genes that are functionally important for heart development and function have come to our attention, including *Tbx3*–*Tbx5*, *Scn5a*–*Scn10a* and *Nppa*–*Nppb*, and have provided insights into the role of structure and composition of the chromatin in genomic function and gene transcription [8, 45, 46].

## Spatial and functional organization of *Nppa*–*Nppb* cluster

With the development of new technologies, different approaches are being used to study loci with respect to their regulatory landscapes of gene loci. These include functional testing of regulatory elements [enhancer and bacterial artificial chromosome (BAC) transgenesis], chromosome conformation capturing, analysis of epigenetic states (ChIP-seq, etc.), and have improved our understanding of the regulatory domains controlling the *Nppa*–*Nppb* cluster [8, 13].

In gene clusters such as *Irx* and *Hox*, the promoters and their shared distal regulatory regions must be brought together physically in order to regulate transcriptional activity. In general, regulatory elements find their target genes within topologically associating domains (TADs). TADs are chromosomal regions, typically about 1 Mbp in size, within which sequences preferentially contact each other. They are separated by boundary regions for CCCTC-binding factor (CTCF) binding sites [47–49]. It has been established that chromatin loops direct enhancers to target genes, thereby creating a three-dimensional regulatory landscape [25, 50, 51]. High-resolution chromatin conformation capturing (4C) revealed that the intergenomic interactions of the *Nppa*–*Nppb* cluster are confined to a domain between the two closest CTCF sites, which is a stretch of approximately 60 kbp [8]. Notably, the chromatin conformation of *Nppa* and *Nppb* differs only little between heart tissue and other tissues, indicating it is permissive, existing in a pre-formed 3D conformation, and not instructive and cell-type dependent [8, 25]. This phenomenon of pre-formed chromatin loops has been demonstrated for other loci as well, including the *Tbx3*–*Tbx5* cluster [52].

Although the exact role of the CTCF sites in *Nppa*–*Nppb* regulation has yet to be investigated, it is thought that CTCF sites maintain the stability of the regulatory domain. Previously it has been described that deletion of CTCF sites in the *Hox* gene clusters (*HoxA* and *HoxD*) disrupted the chromatin conformation and altered the regulatory and transcriptional activities in the TADs [53, 54]. Similarly, changing the orientation of a CTCF site influences DNA-looping interactions, consequently leading to transcriptional misregulation [54, 55]. Recent studies of the functional role of CTCF in chromatin folding and transcriptional regulation describe that CTCF is indeed required for the formation and maintenance of loops between CTCF target sites and architecture of TADs at the genomic level [49, 56, 57]. Conditional depletion of CTCF in mouse embryonic stem cells caused insulation defects at most TAD boundaries and abrogation of chromatin loops between CTCF sites. This resulted in altered enhancer–promoter interactions across the DNA region leading to upregulation of a subset of genes that were previously insulated from neighboring regulatory elements. In addition, it has been suggested that CTCF might also have a direct impact on transcriptional regulation independent of loops and chromatin folding. CTCF sites were often found near transcription start site and were mostly in direct orientation with transcription of the downregulated genes prior to CTCF depletion. In contrast, CTCF depletion did not affect genomic compartments. Restoring CTCF levels reversed the chromatin interactome to its normal state [57].

**Fig. 1:**
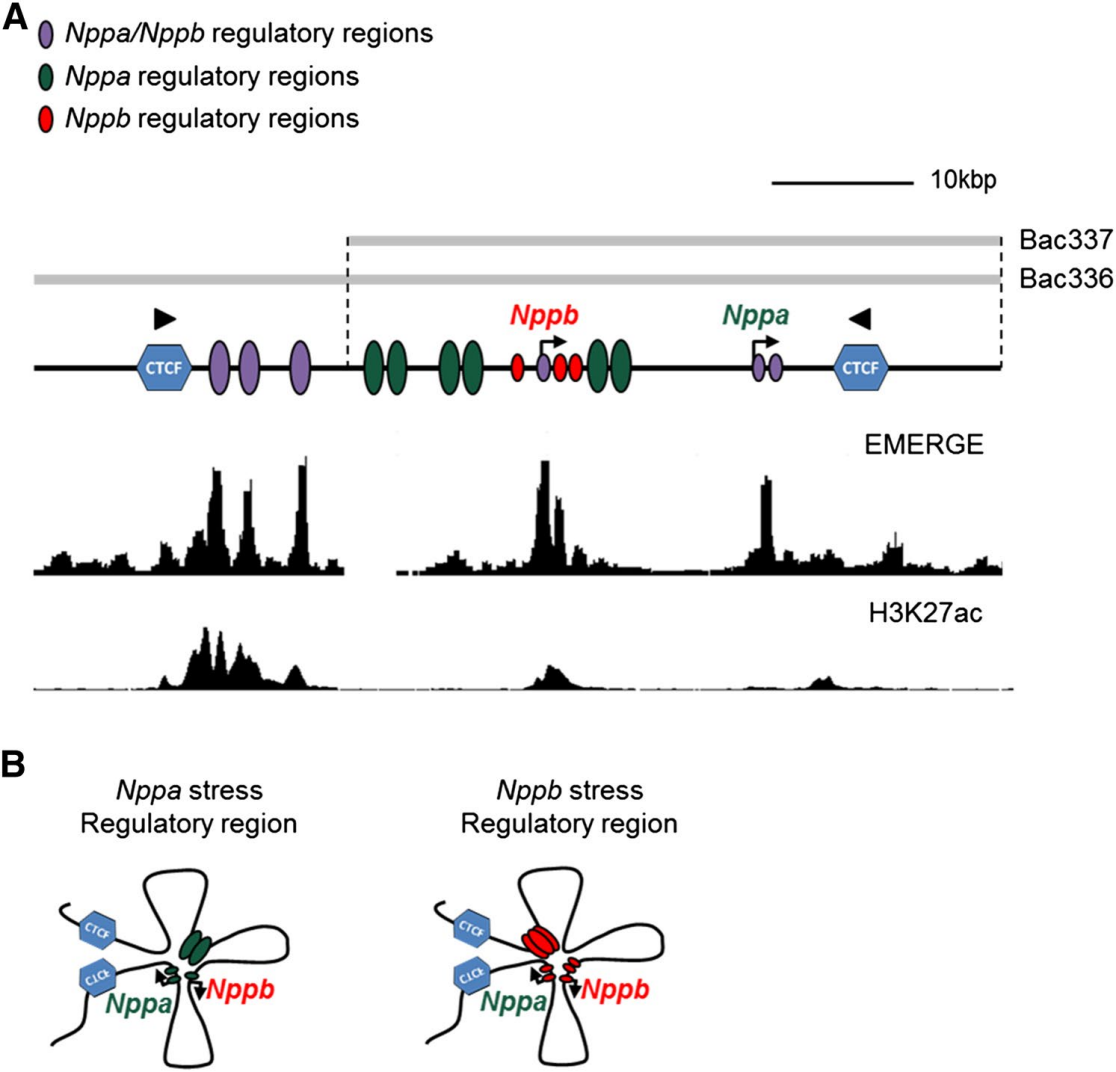
The regulatory landscape of the *Nppa*-*Nppb* locus. **a** Developmental and stress response regulatory regions of *Nppa* and *Nppb* are located within a 60 kbp domain between two CTCF sites arranged in a convergent orientation [49, 91]. Purple, shared regulatory regions of *Nppa*/*Nppb*; green, regulatory regions of *Nppa*; red, regulatory regions of *Nppb*. Gray bars, BAC clones. Displaying EMERGE track for heart and H3K27ac track for mouse cardiomyoyctes [92, 93]. **b** *Nppa* and *Nppb* are regulated by different regulatory elements during stress. The *Nppa* promoter interacts with *Nppb* promoter and several distal and proximal regulatory elements. Stress-induced expression of *Nppb* is regulated by a upstream regulatory region and the *Nppa*/*Nppb* promoters

The accessibility of chromatin relies on structural features, which is tightly controlled by epigenetic processes including DNA methylation, histone modifications and ATP-dependent chromatin remodeling. Particular epigenetic mechanisms are associated with active promoters and *cis*-regulatory elements. In adult cardiomyopathy, including cardiac hypertrophy and heart failure, epigenetic changes such as histone acetylation and methylation are observed in response to cardiac stress. This can contribute to transcriptional reprogramming in the heart and changes in cardiac gene expression [58]. Genome-wide analysis of the epigenetic signature of hypertrophied hearts of mice showed that multiple genes implicated in hypertrophic cardiomyopathy and associated enhancers are modified through histone-3 lysine-27 acetylation (H3K27ac), a modification associated with activation [59]. In patients with heart failure, reactivation of *NPPA* and *NPPB* is correlated with demethylation of H3K9 at their promoter regions, although a modest increase in H3K27ac could also be observed [60]. The cofactor p300, important in acetylation of histones, promotes cardiac remodeling (e.g. left ventricular dilation) in infarcted mouse hearts through interaction with transcription factor Gata4 [61]. Furthermore, p300 is found to be recruited to the *Nppa* and *Nppb* promoter, which is associated with increased histone acetylation such as H3K27ac [62]. Within the *Nppa*-*Nppb* regulatory domain, physical interactions are found between *cis*-regulatory regions and the promoters of *Nppa* and *Nppb*. These regulatory sequences function to control either developmental or stress-responsive expression of *Nppa* and *Nppb*. Analysis of the distribution of H3K27ac and RNA polymerase II (Pol II) across the *Nppa*–*Nppb* locus revealed that epigenetic signatures within the regulatory domain change during cardiac stress. During pressure overload-induced cardiac hypertrophy in mice, H3K27ac is enriched near and at the promoters of *Nppa* and *Nppb*, whereas Pol II occupation, associated with active promoters and enhancers, changed much less. Even though no significant change in Pol II occupation has been observed, both promoters may still be involved in stress-induced expression of *Nppa* and *Nppb*. In the conserved upstream regulatory region that is associated with fetal expression of *Nppa,* the levels of H3K27ac and Pol II are decreased upon stress [7, 8, 63]. It should be noted that in the normal adult heart, this regulatory region is already highly occupied by H3K27ac and presumably maintains *Nppb* expression after birth (Fig. 1a) [8, 59].

## Regulation of the *Nppa*–*Nppb* gene cluster during development and hypertrophy

Genome-wide association studies have found a correlation between genetic variants identified in the *NPPA*–*NPPB* locus and the levels of natriuretic peptides in blood of patients with cardiac dysfunction. A variant (rs5065) in the coding region of *NPPA* [64] and an intronic variant (rs1023252) in *CLCN6* [65] are associated with NT-pro-BNP levels in severe heart failure patients. Furthermore, genetic variants identified upstream and downstream of *NPPB* has proven to significantly affect levels of BNP [66]. Together, this suggest that variants in the *NPPA*–*NPPB* regulatory domain (and in linkage disequilibrium with the reported variants) influence regulatory DNA function. Genetic variants associated with blood pressure and hypertension at the *AGTRAP*–*PLOD1* locus are suggested to influence the expression of multiple genes, including *NPPA* and *NPPB*, within this region [67]. These genetic variants are in linkage disequilibrium with the *NPPA*–*NPPB* regulatory domain and, therefore, may only report the presence of variants influencing regulatory DNA function of *NPPA* and *NPPB* during disease. Indeed, it has been reported that genetic variants positioned within the regulatory domain of *NPPA*–*NPPB* locus potentially affect gene expression by a yet undefined mechanism [68]. The biological and clinical relevance of *Nppa* and *Nppb* is probably the major reason the transcriptional regulation of these genes has been the subject of several studies [7, 8, 22–24]. *Nppa* and *Nppb* are reactivated in the stressed myocardium as part of an induction of a “fetal gene program”. The question remained whether the transcriptional mechanisms involved in hypertrophic stress induction are the same as those governing the fetal gene program. Previously, it has been suggested that the proximal *Nppa* promoter mediates the developmental expression pattern of *Nppa*, although later it was found that its capacity to drive ventricular expression was largely absent [7, 22, 69, 70]. Furthermore, this promoter is inducible in cell culture systems, but not sufficient for stress-induced *Nppa* expression in vivo, suggesting the involvement of other distal regulatory elements [7, 22]. Furthermore, ventricular expression of *Nppa* was found to be driven by distal sequences, whereas stress induction required more proximal sequences, demonstrating that the transcriptional mechanisms driving fetal expression and stress-induced expression are different [7].

The *Nppa* promoter has been used as a model for understanding transcriptional gene regulation during cardiac development [9, 69, 71, 72]. It was thought that the *Nppa* promoter drives embryonic and fetal *Nppa* expression in the atria and ventricles but different fragment sizes of the promoter could not recapitulate the correct ventricular expression [7, 70]. Several regulatory elements that lie upstream of the proximal *Nppa* promoter region appeared to be involved in the ventricular expression of *Nppa* during development. Two reporter BAC clones with 85 kbp of overlapping sequences (BAC336-*EGFP* and BAC337-*EGFP*) (Fig. 1a) covering the *Nppa*–*Nppb* locus were used in an attempt to define the distal regulatory regions that control the pre- and postnatal expression of *Nppa* in the ventricles (Fig. 1a). BAC337-*EGFP* was shown to lack the regulatory sequences necessary for *Nppa* ventricular activity, and unique sequences located in BAC336-*EGFP* (− 141 to – 27 kbp relative to *Nppa*) drove *Nppa*-like expression patterns during development [7]. The potential of this regulatory region (− 141 to – 27 kbp relative to *Nppa*) in mediating the developmental expression of *Nppa* was further supported by analysis of Nkx2–5 occupancy and function in vivo. The transcription factor Nkx2–5 has a major role in the regulation of gene expression in the developing heart. In vivo screening of the regulatory elements within the *Nppa*–*Nppb* locus in inducible Nkx2–5 knockout mice showed a diminished expression in the heart, indicating an essential role of Nkx2–5 in the regulation of *Nppa*. Indeed, 3C analysis showed that these regulatory elements enriched for Nkx2–5 interact with the *Nppa* promoter. However, stress-induced expression of *Nppa* did not depend on Nkx2–5 transcriptional regulation [23]. Further studies on BAC336-*EGFP* and BAC337-*EGFP* revealed that both were able to induce reporter gene expression upon hypertrophic stress. This demonstrates that both BAC clones contain regulatory sequences that mediate stress-induced *Nppa* expression. These sequences are thought to be located in the overlapping 85 kbp region and downstream of *Nppa* (Fig. 1a) [7]. Analysis of both BAC clones revealed that the distal regulatory region is responsible for *Nppa* expression in the embryonic/fetal and adult heart, whereas the proximal regulatory region is required for stress-induced *Nppa* expression.

The development and stress-induced regulatory elements of *Nppb* were less well described compared to *Nppa*. There is evidence that the promoter constitutively drives weak *Nppb* expression in the normal and stressed heart [73, 74]. As described below, later studies showed other regulatory elements within the *Nppa*–*Nppb* locus are required [8].

Recently, a more extensive characterization of the spatial and functional organization of the *Nppa*–*Nppb* cluster in vivo has been provided. Based on H3K27ac and Pol2 ChIP-seq data, heart-specific regulatory regions were defined in the *Nppa*–*Nppb* locus (Fig. 1a), which were functionally tested in a transgenic mouse model carrying a BACs with two modifications. The function of the *Nppa*–*Nppb* cluster can be monitored simultaneously for both *Nppa* and *Nppb* due to the insertion of the *Luciferase* and *Katushka* genes at the translation start sites of these genes, respectively, within the BAC. Both reporter genes recapitulate the tissue-specific and developmental pattern of expression and stress response of endogenous *Nppa* and *Nppb* [8, 13]. Analyses of the BAC transgenic mice showed that developmental expression of *Nppa* and *Nppb* is mediated by shared *cis*-regulatory elements located approximately 27 kbp upstream of *Nppa* (Fig. 1a). This regulatory region, roughly 10 kbp, is enriched for epigenetic features including heart-specific DNaseI hypersensitivity sites and histone modifications, and binding sites for various cardiac transcription factors (e.g. Nkx2–5 and Gata4). Furthermore, this regulatory region is being described as a “super enhancer” [75, 76]. According to the conformation of the *Nppa*–*Nppb* locus, this region contacts the promoters of both genes, suggesting that regulatory elements within this region drive the fetal ventricular expression of *Nppa* and *Nppb*. Furthermore, this region might also contain regulatory elements involved in *Nppb* expression during hypertrophic stress in the adult heart (Fig. 1a, b) A 650-bp fragment located in the same region was implicated in stress-induced *Nppa* expression [24]; however, the BAC transgenesis study indicates it may be involved in *Nppb* regulation. This finding is further supported by analysis of transgenic lines with BAC337 that lacks this region, in which strong *EGFP* expression (reporting for *Nppa*) was observed in stressed ventricles [7, 8, 24]. However, it is uncertain whether this 650 bp fragment is involved in induction during hypertrophy as no response has been observed in vitro after stimulation with phenylephrine (PE) or hypertrophic stress in transgenic mice with this fragment (Sergeeva, unpublished data).

Which particular regulatory elements drive stress-induced *Nppa* expression remains unresolved. There are indications that the *Nppb* promoter might be involved; its deletion within the double reporter BAC rendered *Luciferase*/*Nppa* non-responsive to hypertrophy. However, the *Nppb* promoter alone was not sufficient to drive *Nppa* expression upon hypertrophic stress. The *Nppb* promoter is necessary for the embryonic/fetal and adult expression of *Nppb* itself, but not required for hypertrophic induction. Nevertheless, the *Nppb* promoter drives *Luciferase* expression in rat ventricular cardiomyocytes after PE stimulation. Together, these data suggest that the *Nppb* promoter is part of a complex of proximal and distal regulatory elements, all required in vivo, whereas several of these elements may drive stress-responsive expression when tested outside their endogenous context (Fig. 1a) [8].

## *Nppa*–*Nppb* cluster locus containing conserved regulatory elements activated during zebrafish heart regeneration

Myocardial infarction causes loss of heart muscle. In contrast to lower vertebrates like fish and amphibians, the mammalian heart has a highly insufficient capacity to regenerate and restore this muscle tissue. Studies on zebrafish heart regeneration have demonstrated through genetic lineage tracing that proliferating cardiomyocytes are the source of the newly formed cardiomyocytes. These (adult) cardiomyocytes have first undergone dedifferentiation, which is characterized by disassembly of sarcomeric structures and re-expression of genes such as Gata4 involved in heart development and *Nppa*/*Nppb* [77–80]. Recently is has been shown that a similar regenerative response is found in neonatal mice, in which the cardiomyocytes retain for a short period of time the ability to proliferate [81]. Cardiomyocyte renewal in adult mice (and humans) is very limited under normal conditions with a less turnover rate of less than 1 percent per year [82, 83]. In adult mouse myocardial infarction models, cardiomyocyte proliferation has been observed, but too low to regenerate the injured heart [82, 84, 85].

**Fig. 2:**
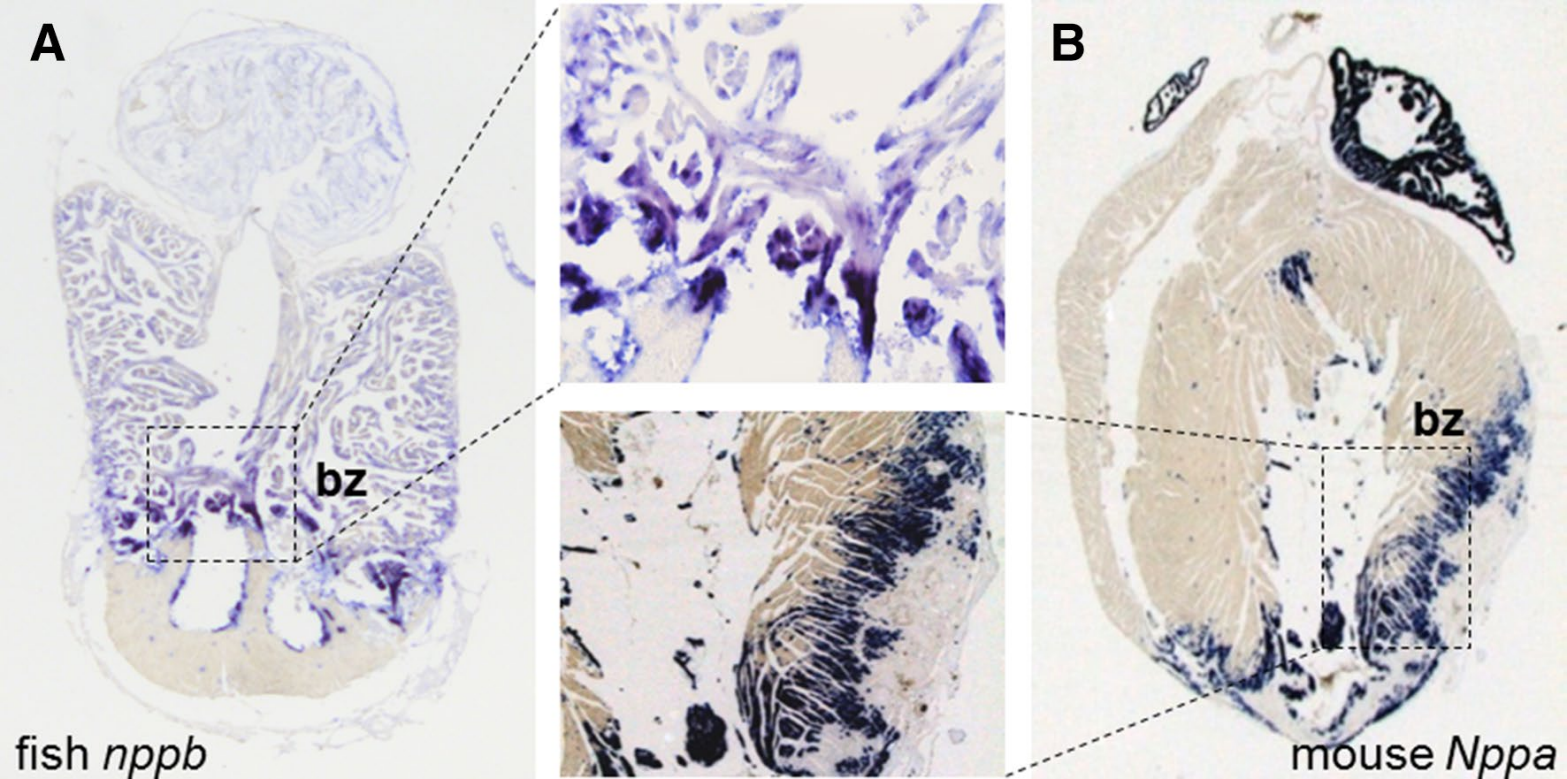
Expression of zebrafish *nppa* and *nppb* and of mouse *Nppa* mRNA in sections of an injured zebrafish and mouse heart. **a**, **b** Both fetal genes are reactivated in the border zone (bz) after cryo-injury and myocardial infarction, respectively [10, 80]

During fetal and neonatal development, cardiomyocytes rapidly proliferate and, therefore, the myocardium can regenerate upon injury. From studies aimed at understanding the regulation of cardiomyocyte proliferation and regeneration it has been suggested that cardiomyocyte-intrinsic programs can promote these regenerative processes upon cardiac injury [86]. Exploiting the transcriptional dynamics during zebrafish heart regeneration suggest that these transcriptional regulatory mechanisms recapitulate the fetal gene program [79, 87]. Furthermore, the spatial gene expression profile of a cryo-injured zebrafish heart revealed the transcriptional activation of *nppa* and *nppb* in a district region (the border zone) within the heart where also regeneration occurs (Fig. 2a) [80]. Interestingly, reactivation of *Nppa* and *Nppb* is also restricted to the border zone of an injured mouse heart (Fig. 2b) [10]. This raises the question whether conserved stress responsive regulatory elements for *Nppa*/*nppa* and *Nppb*/*nppb* exists in the mouse and zebrafish heart that are associated with an intrinsic mechanism for cardiomyocyte renewal. Only recently, evidence suggests that conserved regulatory elements may indeed be present that can induce the transcriptional programs for heart regeneration upon tissue damage. In the zebrafish *leptin b* locus a distal regulatory element has been identified that directs gene expression after injury, including fin amputation and cryo-injury [88]. This regulatory element and response of leptin are not conserved in the mouse, and the regulatory element is active in the endocardium. Nevertheless, the leptin-linked regulatory element was activated in an injured neonatal mouse heart. Furthermore, the leptin-linked regulatory element could activate *Nrg1/ErbB2/ErbB4* pathway to promote cardiomyocyte proliferation after re-sectioning of the zebrafish heart [88]. Recent histone H3.3 replacement profiling of regenerative zebrafish hearts uncovered thousands of putative regenerative-responsive enhancers in the fish genome [89]. These findings raise the possibility that the *Nppa*–*Nppb* cluster might also harbor conserved regulatory elements which are activated after cardiac injury that can initiate transcriptional programs for dedifferentiation and proliferation of adult cardiomyocytes. Studying the transcriptional regulation of *Nppa* and *Nppb* during disease may uncover these regulatory elements.

## Conclusion and future perspectives

The natriuretic peptides ANF and BNP are widely used as biomarkers in various cardiovascular diseases in clinical settings. Studies of the structure and function of the *Nppa*–*Nppb* cluster has provided novel insights into the transcriptional regulatory mechanisms of *Nppa* and *Nppb* expression during heart development and disease. The transcriptional regulation of *Nppa* and *Nppb* has proven to be complex. *Nppa*, which is highly expressed during ventricular stress, is controlled by several different proximal and distal regulatory elements, including the *Nppb* promoter, to regulate its dynamic expression in the embryonic/fetal and adult heart. *Nppb* expression relies on the interaction of its promoter and a conserved large distal regulatory region, classified as a “super enhancer”. Moreover, the *Nppa*–*Nppb* cluster shares (developmental) enhancers found in the super enhancer region. The *Nppa*–*Nppb* gene cluster provides a conceptual framework for understanding gene cluster function and enhancer sharing that likely applies to other loci that harbor clustered genes. Other interesting gene clusters such as *Tbx3*–*Tbx5* [45], *Scn5a*–*Scn10a* [46], *Kcne1*–*Kcne2*, *Kcnj2*–*Kcnj16*, *HoxA* and *HoxB* [90] are being studied or have yet to be studied with respect to transcriptional (co-)regulation and genomic function in the heart. Although the paradigm of heart regeneration in the mammalian adult heart is being debated, evidence suggests that conserved regulatory elements are activated after cardiac injury, which controls the transcriptional programs for heart regeneration in fish. Therefore, an intriguing question is whether the regulatory elements found in the *Nppa*–*Nppb* cluster respond to a regenerative mechanism in the stressed myocardium. Future research may focus on the manipulation of the regulatory sequences of the *Nppa*–*Nppb* locus in vivo by CRISPR/Cas9 genome editing to determine their physiological relevance in the context of hypertrophic stress or ischemic injury. Furthermore, stress response regulatory elements of the mammalian *Nppa*–*Nppb* cluster can be integrated into the zebrafish genome by site-directed transgene integration to assess whether these sequences are transcriptionally activated during zebrafish heart regeneration. The identification of these conserved regulatory elements can provide tools to drive therapeutic genes that promote adult mammalian heart regeneration.

## Abbreviations

ANF: Atrial natriuretic factor
BAC: Bacterial artificial chromosome
BNF: Brain natriuretic peptide
BZ: Border zone
CTCF: CCCTC-binding factor
Irx: Iroquois
PE: Phenylephrine
Pol II: RNA polymerase II
TADs: Topologically associated domains
